# NORTH AMERICAN CONTEXTS USING COMPUTERIZED NETWORKS TO IMPROVE CARE DELIVERY FOR OLDER ADULTS

**DOI:** 10.1093/geroni/igac059.251

**Published:** 2022-12-20

**Authors:** Gregory Alexander

## Abstract

Evidence shows that technology provides a means to improve communication and quality of care, through greater efficiencies in information management. However, research demonstrates that technology use does not consistently improve care. Therefore, there is a continuing need globally to evaluate and discuss the impact of technology in clinical care, especially in settings where older people have higher care needs. The panel includes five interdisciplinary experts with backgrounds in care delivery systems for older adults, engineering, informatics, health systems, quality improvement, mobile health, medicine, and nursing from the U.S. and Canada. This expert panel has three objectives: 1) Describe informatics research initiatives using computerized networks to improve care delivery for older adults, 2) Contrast information technologies used to manage information in health systems caring for older adults, and 3) Explain opportunities to improve health information technology systems used in care delivery for older adults.

